# Activating *PIK3CA* mutations coexist with *BRAF* or *NRAS* mutations in a limited fraction of melanomas

**DOI:** 10.1186/s12967-015-0401-8

**Published:** 2015-01-28

**Authors:** Antonella Manca, Amelia Lissia, Mariaelena Capone, Paolo A Ascierto, Gerardo Botti, Corrado Caracò, Ignazio Stanganelli, Maria Colombino, MariaCristina Sini, Antonio Cossu, Giuseppe Palmieri

**Affiliations:** Institute of Biomolecular Chemistry, National Research Council (CNR), Traversa La Crucca 3 - Baldinca Li Punti, 07100 Sassari, Italy; Department of Pathology, Hospital-University Health Unit (AOU), Sassari, Italy; Istituto Nazionale Tumori, Fondazione Pascale, Naples, Italy; Skin Cancer Unit, Istituto Scientifico Romagnolo Tumori (IRST), Meldola, Italy

**Keywords:** Melanoma, Mutation analysis, *PIK3CA* gene, Resistance to BRAF/MEK inhibitors

## Abstract

**Background:**

Activated PI3K-AKT pathway may contribute to decrease sensitivity to inhibitors of key pathogenetic effectors (mutated BRAF, active NRAS or MEK) in melanoma. Functional alterations are deeply involved in PI3K-AKT activation, with a minimal role reported for mutations in *PIK3CA*, the catalytic subunit of the *PI3K* gene. We here assessed the prevalence of the coexistence of *BRAF*/*NRAS* and *PIK3CA* mutations in a series of melanoma samples.

**Methods:**

A total of 245 tumor specimens (212 primary melanomas and 33 melanoma cell lines) was screened for mutations in *BRAF*, *NRAS*, and *PIK3CA* genes by automated direct sequencing.

**Results:**

Overall, 110 (44.9%) samples carried mutations in *BRAF*, 26 (10.6%) in *NRAS*, and 24 (9.8%) in *PIK3CA*. All identified *PIK3CA* mutations have been reported to induce PI3K activation; those detected in cultured melanomas were investigated for their interference with the antiproliferative activity of the BRAF-mutant inhibitor vemurafenib. A reduced suppression in cell growth was observed in treated cells carrying both *BRAF* and *PIK3CA* mutations as compared with those presenting a mutated *BRAF* only. Among the analysed melanomas, 12/245 (4.9%) samples presented the coexistence of *PIK3CA* and *BRAF*/*NRAS* mutations.

**Conclusions:**

Our study further suggests that *PIK3CA* mutations account for a small fraction of PI3K pathway activation and have a limited impact in interfering with the BRAF/NRAS-driven growth in melanoma.

## Introduction

Several cell-signaling pathways participate in development and progression of melanoma. Among others, two RAS-driven signal-transduction networks play a crucial role in melanoma pathogenesis: the PI3K/AKT/mTOR and, mainly, the BRAF/MEK/ERK pathways [1–3]. Activation of the latter one, also known as the *mitogen-activated protein kinase* (MAPK) pathway, is mostly driven by oncogenic mutations in *BRAF* and, to a less extent, in *NRAS* genes; somatic mutations in such two genes are mutually exclusive and able to stimulate cell proliferation and tumor growth, through induction of a constitutive ERK phosphorylation [1–3]. Different events may instead contribute to activate the PI3K/AKT pathway: PI3K stimulation by active GTP-bound RAS, occurrence of activating mutations in *PIK3CA* (the catalytic subunit of the *PI3K* gene), or silencing of the *PTEN* tumor suppressor gene [4,5]. The intracellular accumulation of active AKT results in enhancement of cell survival, migration capability, and resistance to apoptosis in human cancers, including melanoma [6,7].

At present, inhibitors of key effectors into the MAPK pathway (BRAF-mutant inhibitors, as vemurafenib or dabrafenib, MEK inhibitors, as trametinib, and their combination) are allowing to overcome the ineffectiveness of the conventional therapies [8]. In patients treated with such inhibitors, a rapid acquisition of drug resistance, as consequence of reactivation of the MAPK pathway or activation of alternative signaling pathways, has been reported to however limit the survival benefits [9,10]. Nevertheless, a fraction of them are primarily refractory due to an intrinsic resistance to such inhibitors [9,10]. On this regard, an increasing amount of evidence indicates that multiple mechanisms may contribute to the development of resistance in melanoma, including those underlying intratumor heterogeneity, alterations in tumor microenvironment (i.e. growth factors and cytokines that interact with their corresponding receptors as well as hormones and neuropeptides), and the ability of tumor to generate an immunosuppressive environment [3,8,11–13]. Even different levels of intralesional pigmentation may interfere with melanoma pathogenesis and/or affect the behavior of the disease [12,13].

At intracellular and molecular level, crosstalk mechanisms between the MAPK and PI3K/AKT pathways, with the mutated BRAF inducing a negative regulation of the AKT network, have been described [14]. Inactivation of the oncogenic BRAF by targeted inhibitors is thus supposed to increase the intracellular levels of phosphorylated AKT, contributing to the enhancement of cell survival and the development of drug resistance [14]. Suppression of AKT activity by inhibition of either upstream (PI3K) or downstream (mTOR) effectors of this signaling cascade is being proposed as an effective tool for the improvement of the antitumor response to the MAPK-targeted therapies [15–17]. In preclinical studies, combined treatment based on inhibition of BRAF and silencing of AKT3 was found to significantly increase suppression of tumour growth as compared to the result obtained by single agent administration [18,19]. Recently, combination of a BRAF or MEK inhibitor with a PI3K/mTOR inhibitor was found to enhance cell growth inhibition through achievement of ERK hypo-phosphorylation, overcoming the resistance encountered by the use of a single anti-BRAF or anti-MEK agent [17,20].

Overall, identification of melanomas with activated alternative signaling pathways may be helpful in selecting the fraction of patients carrying *BRAF* mutations primarily refractory to the treatment with either a BRAF or MEK inhibitor. In our case, this raises the question whether a test for detecting the activation of the PI3K/AKT pathway should be routinely used in clinical practice for a more accurate classification of the patients before addressing them to be treated with such inhibitors.

Toward the identification of a more appropriate test for assessing the activated status of the PI3K/AKT pathway, genetic variations, whose assessment is qualitative (detecting the objective presence or absence of each specific sequence variant), can be considered as more reliable predictive markers as compared to expression alterations, whose classification is quantitative or semi-quantitative (for immunohistochemistry, strictly depending on subjective evaluation of both intensity and distribution of tissue protein staining). Since *PTEN* is mostly inactivated by gene deletions or rearrangements [21] as well as *AKT* and *mTOR* are mainly altered at functional level, *PI3K* - and, particularly, its catalytic subunit *PIK3CA* - remains the gene mostly affected by activating somatic mutations into this pathway [5].

The aim of this study was to investigate the prevalence and distribution of pathogenetic variants in *BRAF*, *NRAS*, and *PIK3CA* genes among 245 DNA samples from pigmented melanomas of cutaneous origin, defining the fraction of cases harboring coexistent *PIK3CA* and *BRAF* or *NRAS* mutations. Melanoma cell lines with coexistence of *PIK3CA* and *BRAF* mutations were also investigated to evaluate the level of interference with the anti-proliferative effects of the BRAF-mutant inhibitor vemurafenib.

## Methods

### Samples

One hundred and eighty-six patients with histologically-proven diagnosis of pigmented melanoma of cutaneous origin were included into the study. After obtaining their written consent for tissue sampling, patients were enrolled consecutively between March 2010 and November 2012 from centers in Italy, regardless of cancer family history and disease characteristics. Formalin-fixed, paraffin-embedded primary melanoma tissues were obtained from pathological archives for all patients; in 26 of them with multiple primary melanoma, two synchronous or asynchronous primary tumor tissues were collected. Synchronous melanomas were diagnosed in four patients during the same first observation or, at the most, within one month from the first diagnosis, according to previously defined criteria [22]. In the remaining 22 patients with asynchronous multiple melanomas, the subsequent primary tumors were diagnosed at a median time from the first diagnosis of 28 months (range, 6–83 months). Vast majority (189/212; 89%) of primary melanomas included into the study were from intermittently sun-exposed skin.

To improve sensitivity of nucleotide sequencing (sequence variants can be detected when the mutant alleles are at least 15%-20% of the analyzed DNA sample), the neoplastic portion of each tissue section was isolated in order to obtain tumor samples with at least 80% neoplastic cells.

### Mutation analysis

Genomic DNA was isolated from tumor tissues, using standard methods. The coding sequence and splice junctions of the mostly mutated domains of candidate genes (exon 15 in *BRAF*, exons 2 to 4 in *NRAS*, and exons 9 and 20 in *PIK3CA*) were screened for mutations by directly sequencing the amplified PCR products, using an automated fluorescence-cycle sequencer (ABIPRISM 3130, Life Technologies/ThermoFisher Scientific, Waltham, MA, USA). Primer sequences were as previously-reported by our group [23,24]. Sequencing analysis was conducted in duplicate (two PCR assays from two different tumor sections) and in both directions (forward and reverse) for all samples. A nucleotide sequence was considered as valid when the quality value (QV) was higher than 20 (<1/100 error probability); in this study, the QV average was 40 (range, 30–45; <1/1000-1/10,000 error probability).

### Melanoma cell lines and in vitro proliferation test

For *in vitro* proliferation assay, four melanoma cell lines were selected: 13443-Mel (with BRAF^wild-type^ and PIK3CA^wild-type^; as negative control), PNP-Mel (with BRAF^V600E^ and PIK3CA^wild-type^), M14 (with BRAF^V600E^ and PIK3CA^P539R^), and M259 (with BRAF^V600E^ and PIK3CA^E545K^). Cells were plated in triplicate in 96-well plates, at a density of 3–5 × 10^3^ per well, in fresh medium (RPMI 1640; Invitrogen/ThermoFisher Scientific, USA) only, as control, or medium containing different concentrations of vemurafenib (PLX4032, RG7204; Selleck Chemicals, Houston, TX). In particular, melanoma cell lines were treated in triplicate with increasing concentrations (5 to 100 nM) of vemurafenib for 72 hrs. The percentage of melanoma cell proliferation was estimated on day 4 by a colorimetric assay, as we previously described [25].

### Statistical analysis

All *in vitro* data derive from at least three independent experiments and results are expressed as mean values with 95% confidence intervals. The statistical significance of differential findings between experimental and control groups was determined by ANOVA with the Tukey’s multiple comparison test in Graph-Pad Prism 3.0 software (Graph-Pad Software, Inc., San Diego, CA, USA). These findings were considered significant if two-tailed P values were <0.05.

## Results and discussion

A total of 245 tumor specimens (212 primary melanomas and 33 melanoma cell lines) was screened for mutations in *BRAF*, *NRAS*, and *PIK3CA* genes. Tumor tissues were collected from 186 patients, since 26 of them presented two synchronous or asynchronous primary melanomas (see Methods for details). Median age of the enrolled patients was 51 years (range, 19–83 years); 99 (53%) were men. Patients’ characteristics are shown in Table 1. For majority (18/33; 55%) of melanoma cell lines, main genotypic features have been previously described [26]; however, an extensive genetic and molecular analysis of candidate genes among the entire series of cell lines is being completed (Sini, manuscript in preparation).

**Table 1:** **Characteristics of analyzed patients and melanomas**

**Characteristics**	**No.**	**%**
***Total patients analyzed***	186	
*Males/Females*	99/87	53/47
Median age (years)	51	
Range	19-83	
*Single/Multiple melanoma*	160/26	86/14
*Patients’ AJCC stage*		
I	39	21
II	76	41
III	47	25
IV	24	13
***Total melanoma analyzed***	212	
*Primary site*		
Head and neck	35	17
Limbs	79	37
Trunk	98	46
*Types*		
Superficial spreading	135	64
Nodular	77	36

AJCC, American Joint Committee on Cancer.

Overall, 160 (65.3%) melanoma samples carried mutations in at least one of such candidate genes: 110 (44.9%) in *BRAF*, 26 (10.6%) in *NRAS*, and 24 (9.8%) in *PIK3CA* (Table 1). For *PIK3CA* mutations, screening revealed the occurrence of five mutations (p.P539R, p.E542K, p.E545A, p.E545G, and p.E545K in exon 9; no sequence variation was detected in exon 20), which have been widely reported in mutation databases [Human Gene Mutation Database (HGMD) at http://www.hgmd.cf.ac.uk/ac/all.php and Catalogue Of Somatic Mutations In Cancer (COSMIC) at http://cancer.sanger.ac.uk/cancergenome/projects/cosmic/] as commonly associated with human cancer, with a recognized functional role of the corresponding mutated proteins. The variant p.E545A was the mutation with the highest frequency in our series [detected in 14/24 (58.3%) *PIK3CA* mutants].

Among analyzed melanomas, 12/245 (4.9%) samples presented the coexistence of *PIK3CA* and *BRAF* or *NRAS* mutations [11 cases with *BRAF* and *PIK3CA* mutations and 1 case with *NRAS* and *PIK3CA* mutations; Table 2). On the other hand, *NRAS* or *BRAF* mutations were found in half (12/24; 50%) of patients with *PIK3CA* mutations, which were conversely detected in less than one tenth (12/136; 8.8%) of cases with *NRAS* or *BRAF* mutations (Table 2). Among the 212 tumor tissues of our series, 52 samples were from 26 patients with multiple primary melanoma (all such cases presented two synchronous or asynchronous primary melanomas). As shown in Table 3, about half (12/26; 46.2%) of patients with multiple melanoma showed discrepancies in mutation patterns between first and subsequent primary tumors, further supporting our previous observations that molecular mechanisms underlying the development of multiple melanomas in the same patients are heterogeneous [27,28].

**Table 2:** **Frequencies of somatic mutations in the three candidate genes**

**Sample**	***BRAF***	***NRAS***	***BRAF + PIK3CA***	***NRAS + PIK3CA***	***PIK3CA***	***Wild-type***
	***%***	***%***	***%***	***%***	***%***	***%***
Primary melanoma (N = 212)	81	21	9	1	10	90
	*38.2*	*9.9*	*4.2*	*0.5*	*4.7*	*42.5*
Melanoma cell line (N = 33)	18	4	2	0	2	7
	*54.5*	*12.1*	*6.1*	*0*	*6.1*	*21.2*
Total (N = 245)	99	25	11	1	12	97
	*40.4*	*10.2*	*4.5*	*0.4*	*4.9*	*39.6*

**Table 3:** **Consistency between** ***BRAF/NRAS/PIK3CA*** **mutations in multiple melanomas from same patients, and mutation patterns in those in whom there were discrepancies**

**No. of cases**	**Cases with consistent mutation patterns (second** ***vs*** **first tumor sample), n (%)**	**Mutation patterns among discrepant paired samples**					
		***BRAF***		***NRAS***		***PIK3CA***	
		**First tumor**	**Second tumor**	**First tumor**	**Second tumor**	**First tumor**	**Second tumor**
26	14 (53.8)	wt	V600E	wt	wt	wt	wt
		wt	wt	wt	wt	wt	E545A
		V600E	wt	wt	Q61R	E545A	wt
		V600K	wt	wt	wt	wt	wt
		wt	wt	wt	wt	E545A	wt
		V600E	wt	wt	wt	wt	wt
		wt	wt	wt	wt	wt	E542K
		V600E	wt	wt	wt	wt	wt
		V600E	wt	wt	wt	wt	wt
		wt	wt	Q61R	wt	wt	wt
		wt	V600E	wt	wt	E545G	wt
		wt	V600E	wt	wt	wt	wt

*Abbreviation*: *wt* wild-type.

**Figure 1:**
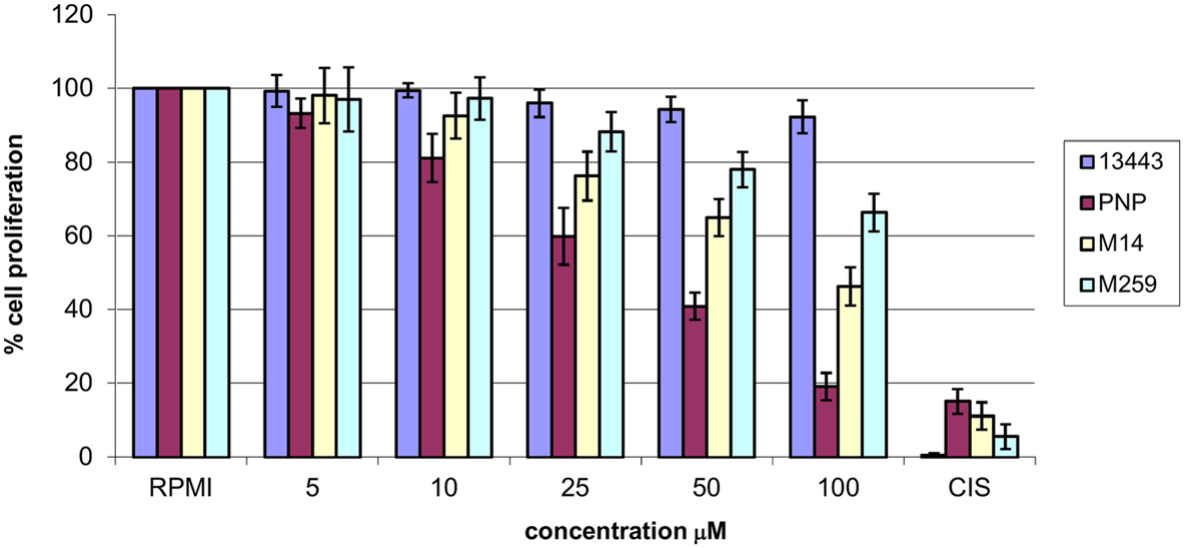
**Effects of vemurafenib on the growth of human melanoma cell lines.** Four melanoma cell lines were cultured in presence of various concentrations of vemurafenib for 72 hours and cell proliferation was estimated as described in Methods. Results are expressed as percent of cell growth and represent the average (± standard deviation) of triplicate experiments. RPMI, cell cultures in fresh medium only, as control. Cisplatinum (CIS) at concentration of 5 μM was used as cytotoxicity positive control.

Using a panel of four melanoma cell lines carrying constitutive differences in mutational status of both *BRAF* and *PIK3CA* genes (including the only two cell lines with coexistence of *BRAF* and *PIK3CA* mutations; see Table 2 and Methods), we have investigated the ability of the BRAF-mutant inhibitor vemurafenib to halt cell proliferation. As expected, a remarkable anti-proliferative activity by this drug was observed in the PNP-Mel cells (presenting a mutated *BRAF* and a wild-type *PIK3CA*), whereas lack of a significant inhibitory effect on growth was demonstrated in control cells carrying wild-type *BRAF* and *PIK3CA* (Figure 1). Suppression in cell proliferation was instead less evident or minimal on *BRAF*-mutated M14 (presenting the p.P539R mutation in *PIK3CA*) and M259 (with the p.E545K mutation in *PIK3CA*) cell lines, respectively (Figure 1). Although experimental assays should be carried extensively on a larger series of melanoma cell lines, our findings permit to speculate that activating mutations of *PIK3CA* may interfere with the anti-proliferative effects of BRAF inhibitors on melanomas carrying oncogenic variants of *BRAF* (though levels of interference may vary, strictly depending on the type of sequence alterations at the functional domains of the *PIK3CA* gene). Although our results are consistent with data from literature - indicating that absence of an activated PI3K-AKT pathway may contribute to increase sensitivity to drugs inhibiting the oncogenically-active MAPK components (either mutated BRAF or MEK) [29–32], it is to keep in mind that other molecular features (BRAF amplification, BRAF splice variants, MEK1-2 mutations, RTK/NRAS activation, PTEN inactivation, RB1 loss, etc.) may be associated with the decreased sensitivity to BRAF inhibitors [10,11,33].

In our study, prevalence of pathogenetic *PIK3CA* mutations in cutaneous melanoma tissues (20/212; 9%) was much higher than that (about 2%) reported in HGMD and COSMIC databases for *in vivo* melanoma samples. Considering a collection of about 1,000 melanoma samples from the most relevant studies published on this issue [34–38], mutation rates in *PIK3CA* gene were consistently identical during melanoma progression - from primary cutaneous melanomas (2.3%) to metastatic melanomas (2.5%) and melanoma cell lines (2.5%). For *in vitro* melanoma samples from our series, frequency of *PIK3CA* mutations (4/33 melanoma cell lines; 12%) was even higher than that above reported (though *BRAF*/*NRAS* mutations remained the most prominent genetic alterations, acting as main drivers of proliferation in vast majority of cultured melanomas). Conversely, the rate of coexistence of *PIK3CA* and *BRAF* or *NRAS* mutations (about 5%) in our series was lower than that previously described (20%; [4]).

Overall, our findings further confirm that *PIK3CA* is rarely subject to somatic activation through genetic alterations in melanoma. In other words, sequence variations in *PIK3CA* gene only account for a small fraction of activations of the PI3K-AKT signaling pathway and are thus expected to minimally interfere with the *BRAF*/*NRAS*-driven growth. Conversely, activation of AKT, the main downstream component of the PI3K pathway, is mostly determined at functional level as consequence of an increased activity of the upstream effectors (RAS activation, PTEN suppression), with again a minimal role played by genetic alterations (activating mutations in *AKT1* and *AKT3* genes have been reported in a limited number of melanomas and melanoma cell lines) [14,30,39]. Although it is becoming evident that multiple mechanisms may contribute to AKT activation in melanoma, loss of PTEN - reported in about 10% of melanoma tissues and 30% of cultured melanoma cells [35–37] - is considered the major mechanism for abrogating oncogene-induced senescence in either *BRAF* or *NRAS* mutant cells through activation of the PI3K-AKT signaling pathway [6,9,40,41]. In our collection of 33 melanoma cell lines, loss of the PTEN expression was found in eight (24%) of them by immunocytochemistry (data not shown). For this reason, occurrence of PTEN inactivation should be mainly evaluated in melanoma patients harboring activation of the MAPK pathway and undergoing treatment with BRAF and MEK-selective inhibitors in order to determine whether these alterations may be associated with a diminished clinical benefit.

In very next future, more accurate molecular signatures of cancer tissues will be achieved through the advancements of the technologies. Indeed, next-generation sequencing (NGS) approaches are already being demonstrated to better characterize molecular mechanisms underlying resistance to targeted therapies [33,42]. As consequence, more reliable molecular biomarkers might be available for improving the accurateness of the prediction of the treatment outcome among patients with melanoma.

## Conclusions

In our study, the presence of a limited but representative fraction of cases with coexistence of somatic mutations in *PIK3CA* and *BRAF* or *NRAS* genes seems to somehow represent an indicator for including mutation analysis of *PIK3CA* into the initial (prior-to-therapy) strategies for molecular classification of melanoma patients. This could allow to prospectively make correlations between the coexistence of *PIK3CA*/*BRAF*/*NRAS* mutations and rates of intrinsic resistance to BRAF or MEK inhibitors.

## Abbreviations

COSMIC: Catalogue of somatic mutations in cancer
HGMD: Human gene mutation database
MAPK: Mitogen-activated protein kinase
NGS: Next-generation sequencing
PCR: Polymerase chain reaction
